# Oncolytic adenovirus targeting cyclin E overexpression repressed tumor growth in syngeneic immunocompetent mice

**DOI:** 10.1186/s12885-015-1731-x

**Published:** 2015-10-16

**Authors:** Pei-Hsin Cheng, Xiao-Mei Rao, Stephen L. Wechman, Xiao-Feng Li, Kelly M. McMasters, Heshan Sam Zhou

**Affiliations:** 1 Department of Pharmacology and Toxicology, University of Louisville School of Medicine, Louisville, KY 40292 USA; 2 James Graham Brown Cancer Center, University of Louisville Medical School, 505 South Hancock Street, CTR Building, Room 306, Louisville, KY 40202 USA; 3 Department of Diagnostic Radiology, University of Louisville School of Medicine, Louisville, KY 40292 USA; 4 Hiram C. Polk Jr MD Department of Surgery, University of Louisville School of Medicine, Louisville, KY 40292 USA; 5 Department of Microbiology and Immunology, University of Louisville School of Medicine, Louisville, KY 40292 USA

**Keywords:** Adenovirus, Oncolytic virotherapy, Cyclin E, Immunocompetent model, Lung cancer

## Abstract

**Background:**

Clinical trials have indicated that preclinical results obtained with human tumor xenografts in mouse models may overstate the potential of adenovirus (Ad)-mediated oncolytic therapies. We have previously demonstrated that the replication of human Ads depends on cyclin E dysregulation or overexpression in cancer cells. ED-1 cell derived from mouse lung adenocarcinomas triggered by transgenic overexpression of human cyclin E may be applied to investigate the antitumor efficacy of oncolytic Ads.

**Methods:**

Ad-cycE was used to target cyclin E overexpression in ED-1 cells and repress tumor growth in a syngeneic mouse model for investigation of oncolytic virotherapies.

**Results:**

Murine ED-1 cells were permissive for human Ad replication and Ad-cycE repressed ED-1 tumor growth in immunocompetent FVB mice. ED-1 cells destroyed by oncolytic Ads in tumors were encircled in capsule-like structures, while cells outside the capsules were not infected and survived the treatment.

**Conclusion:**

Ad-cycE can target cyclin E overexpression in cancer cells and repress tumor growth in syngeneic mouse models. The capsule structures formed after Ad intratumoral injection may prevent viral particles from spreading to the entire tumor.

**Electronic supplementary material:**

The online version of this article (doi:10.1186/s12885-015-1731-x) contains supplementary material, which is available to authorized users.

## Background

Virotherapy with *E1b55K*-deleted adenoviruses (Ads), which preferentially replicate in cancer cells causing oncolysis and amplified efficacy, has been considered as an emerging drug platform [1]. Although *E1b55K*-deleted ONYX-015 (dl1520) has been applied in clinical trials and H101 (structurally similar to ONYX-015) has been commercially approved for cancer treatment [2, 3], the antitumor effects of oncolytic Ads have been somewhat disappointing in clinical applications [4, 5].

Clinical studies have indicated the importance of developing better preclinical tumor models [1]. Current animal models used to evaluate the efficacy of oncolytic Ads rely on human tumor xenografts in immunodeficient mouse models. Nevertheless, such kind of human tumor xenograft models are less clinically relevant. The lack of functional immune systems in immunodeficient models may hinder the accuracy of predicting the clinical effects in human patients [6, 7]. Ads have complex interactions with host immune response effectors [2, 8]. In the presence of the immune system, the oncolytic effects of the virus may be reduced due to the immune responses against viral particles. Conversely, the immune system may rally round the tumor-killing effects by recruiting natural killer cells, antibodies, or tumor-specific cytotoxic T lymphocytes (CTLs) to enhance the therapeutic outcome [9, 10]. Additionally, human Ads tend to infect and replicate more efficiently in human cancer cells than in normal murine tissues in the mouse models. Thus, preclinical results obtained with human tumor xenografts in mouse models may overstate the therapeutic potential. In fact, results of clinical trials often fall short of hopes and expectations based on preclinical animal studies. It has become clear that the development of suitable immunocompetent murine cancer models for studies of Ad-mediated oncolysis will benefit the evaluation of virotherapies in more clinically relevant settings.

It was originally proposed that the *E1b55K*-deleted Ads could replicate only in p53-deficient tumor cells, as the E1B55K-mediated degradation of p53 protein was not required in those cancer cells [11, 12]. However, the original hypothesis was challenged by several studies showing that *E1b55K*-deleted Ads are able to replicate in cells regardless of their p53 status [13–16]. Previously, we have demonstrated that cyclin E dysregulation or overexpression in cancer cells is an important molecular basis of selective replication of *E1b55K*-deleted Ads in human cancer cells [17, 18]. Wild-type Ad infection induces cyclin E overexpression in normal and cancer cells. *E1b55K*-deleted Ads fail to efficiently induce cyclin E in normal cells, and thus viral replication is restricted; however, *E1b55K*-deleted Ads can efficiently induce cyclin E in cancer cells with dysregulated cyclin E and successfully replicate in these cancer cells. We have reported that Ad-induced cyclin E activates CDK2 and targets the transcriptional repressor pRb that may affect the cellular environment for viral productive replication [19].

Cyclin E is a nuclear protein essential for the cell cycle progression [20], DNA replication [21, 22], and centrosome duplication [23, 24]. Numerous types of cancers are highly associated with dysregulation of cyclin E [25]. Dysregulation of cyclin E occurs in more than 90 % of lung, liver, and gastrointestinal cancers, and in more than 80 % of glioma/blastoma, bone, and breast cancers [26]. Constitutive overexpression of cyclin E induces chromosome instability [27, 28], impairs normal cell cycle progression, and triggers tumor development in transgenic animal models [29–31]. Human cyclin E overexpression in mouse lungs lead to the development of premalignant and malignant lung lesions that resemble the features found in lung cancer patients [31, 32]. A murine ED-1 cell line was derived from lung cancers of cyclin E transgenic mice [32, 33].

We have developed a novel *E1b*-deleted oncolytic Ad vector, Ad-cycE, in which the *E1a* gene is under the control of the human cyclin E promoter [34]. With the deletion of entire *E1b* region, Ad-cycE shares the replication pattern similar to *E1b55K*-deleted dl1520 which relies on the cyclin E overexpression in cancer cells. As the cyclin E promoter is highly active in multiple types of cancer cells and would be further stimulated after Ad infection, Ad-cycE replication could be enhanced in cancer cells. We showed that Ad-cycE elicits efficient antitumor effects not only in cancer cells reported as permissive for dl1520 replication but also in those reported as non-permissive for dl1520. Ad-cycE significantly repressed tumor growth in the immunodeficient nude mice bearing human lung cancer xenografts. In this study, we aimed to evaluate the impact of Ad-cycE in a more clinical relevant model. We compared and characterized the replication pattern of oncolytic Ads in human and murine lung cancer cells. Our results showed that the ED-1 murine cancer cells are permissive for human Ad replication, and that Ad-cycE significantly represses ED-1 tumor growth in immunocompetent mice. The availability of this syngeneic model will allow the opportunity to study the interaction between oncolytic viruses and the immune system. Our model may provide a better preclinical system to evaluate virotherapeutic efficacy, safety, pharmacokinetics, and vector biodistribution.

## Methods

### Cell lines and culture conditions

HEK 293 (ATCC no. CRL-1573), human lung cancer A549 (ATCC no. CCL-185), and mouse embryonic fibroblast NIH/3T3 (ATCC no. CRL-1658) cell lines were purchased from the American Type Culture Collection (Rockville, MD). The murine ED-1 cell line, a lung cancer cell line derived from transgenic mice with wild-type human cyclin E under control of the human surfactant C (SP-C) promoter [32, 33], was a gift from Dr. Ethan Dmitrovsky's lab. HEK 293 and A549 cells were cultured in minimal essential medium Alpha. ED-1 cells were cultured in RPMI-1640 medium. All media were supplemented with 10 % fetal bovine serum (FBS) and penicillin/streptomycin (100 U/ml). Cells were cultured in a 5 % CO_2_ incubator at 37 °C.

### Adenoviral vectors

Wild-type Ad type 5 (Adwt, ATCC no. VR-5) was used as a replication-competent control. AdCMV/GFP, an Ad vector with *E1* deletion carrying a green fluorescent protein (GFP), was used as a replication-defective control. Ad dl1520 is a *E1b* mutant that contains an 827-bp deletion and a point mutation to generate a premature stop codon in the E1B55K coding region [35]. Ad-cycE is a novel *E1b*-deleted oncolytic vector carrying a human cyclin E promoter driving an intact E1A expression cassette [34]. All of the vectors created and used in this study are based on the backbone of wild-type Ad type 5.

### MTT assay

Cell proliferation was assessed at three days after respective treatments by measuring the conversion of the 3-(4,5-dimethylthiazol-2-yl)-2,5-diphenyltetrazolium bromide (MTT) to purple formazan, as previously described [36]. The experiments were repeated at least three times. The results were expressed as the fold change relative to the result at day 0. Doubling time was analyzed from the cell growth curves on log phase with the exponential regression analysis provided by http://www.doubling-time.com/compute.php [37, 38].

### Cytotoxicity assay

Cytotoxicity was assessed with crystal violet staining, as previously described [39]. The OD values were quantitated into the cell viability percentage by the equation: cell viability % = (OD value of experimental group / OD value of control group) × 100 %. The mock-control group was calculated as 100 % of cell viability in the assay [40].

### Southern blot analysis

After viral infection, cells were collected at different time points. The viral DNA synthesis was determined with Southern blot analyses, as described previously [17, 19]. The blot was pre-hybridized for 3 h at 63 °C. The hybridization and stringency washes were performed at 60 °C and followed by the chemiluminescent detection, according to the manufacturer’s protocol. Densitometric value for the bands was quantified by Gel-pro Analyzer 4.0 software (Media Cybernetics, Bethesda, MD) [41] and expressed as integrated optical density (I.O.D.).

### Western blot analysis

Infected cells were harvested at indicated time points and Western blot analyses were performed as described previously [19, 42]. The primary antibodies used in this study were rabbit anti-cyclin E (M-20), (Santa Cruz Biotechnology, Santa Cruz, CA), mouse anti-Ad type 5 E1A (BD Pharmingen, San Jose, CA), and rabbit anti-Ad type 5 antibody (Abcam, Cambridge, MA). The membranes were then incubated with anti-mouse immunoglobulin G (IgG) or anti-rabbit IgG peroxidase-linked species-specific whole antibody (GE Healthcare, Piscataway, NJ).

### Viral titration

Total infected cells and culture supernatants were collected at the indicated time points and lysed to release virus particles with three cycles of freezing and thawing. The viral titers were determined by the infective unit method, as described previously [43, 44]. Briefly, HEK 293 cells were seeded in 96-well plates at a density of 10^3^ (cells/well) and then infected with 10-fold serially diluted viruses. Cytopathic effect (CPE) was recorded and scored after incubation for 7 days.

### Burst assay

Burst assays were used to determine the replication efficiency of human Ads in infected cells [45–47]. Cells were seeded in 6-well plates at a density of 2 x 10^5^ (cells/well) for 4 h and infected with human Ads at 3.5 (for A549 cells) or 10 multiplicity of infection (MOI) (for ED-1 cells). At 18 h post-infection (p.i.), cell supernatants were removed, and the cell monolayers were washed twice with phosphate buffered saline (PBS). At 18 h and 120 h p.i., cells and supernatants were collected. The viral titers were determined by the infective unit method. The burst ratio was expressed as the titer of virus at 120 h p.i. (virus output) relative to the titer of virus at 18 h p.i. (virus input). An increased ratio in virus titer after 120 h indicates virus replication.

### Syngeneic subcutaneous murine lung cancer study

Female FVB/NCr mice were obtained from National Cancer Institute (Bethesda, MD). 5 x 10^6^ ED-1 murine lung cancer cells were subcutaneously injected into the flanks of mice (age, 6 weeks). Once tumor volume reached approximately 50 mm^3^, the mice were randomized and received 1.5 × 10^9^ IFU of AdGFP or Ad-cycE in 50 μL of PBS every 2 days for a total of 4 treatments. The tumors were measured every 3 days; the volume was determined by externally measuring in 2 dimensions with a caliper and calculated based on the following equation: V = (L × W^2^) / 2, where L is length and W is width of the tumor. Animal experiments were performed according to the institutional guidelines approved by the University of Louisville Institutional Animal Care and Use Committee.

### Histological and immunohistochemical analyses

Tumors were harvested one week after the fourth treatment, embedded in optimal cutting temperature compound (O.C.T.) (Sakura Finetek, Torrance, CA), and stored at −20 °C. The sections (8 μm) were subjected to either hematoxylin-eosin (H&E) or immunohistochemical staining (IHC) as described previously [48]. For IHC staining, the sections were incubated with goat-anti-Ad polyclonal antibody (AB1056, Millipore, Billerica, MA) and diluted (1:800) for 1 h at room temperature. The signals were amplified by a biotinylated anti-goat IgG diluted (1:200) in conjunction with VECTASTAIN avidin-biotin complex method kit (Vector Laboratories, Burlingame, CA). Visualization was achieved using 3,3-diaminobenzidine tetrahydrochloride (ImmPACT DAB peroxidase substrate, Vector Laboratories). Hematoxylin was used as a counterstain. Images were acquired at X200 magnification by using an Olympus BX53 microscope (Olympus, Center Valley, PA).

### Statistical analyses

Quantitation results were reported as means ± standard deviation (S.D.). Statistical differences of the combination experiment were assessed with a Student's *t*-test. Statistical significance of difference was set at *p* < 0.05.

## Results

### Murine ED-1 cells show higher growth rate and lower serum requirement than human A549 cells

**Fig. 1:**
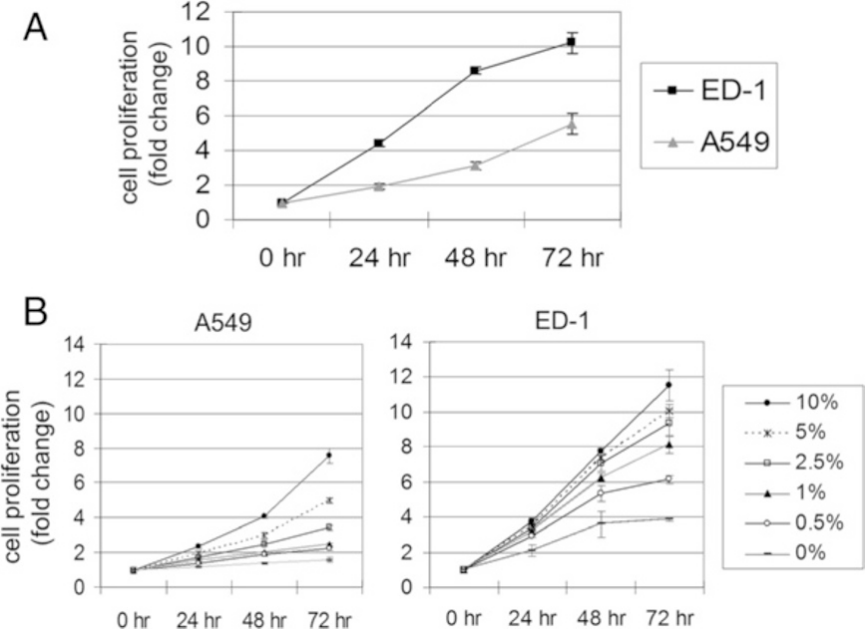
Growth properties of human A549 compared with murine ED-1 cells. Cell proliferation and serum sensitivity of A549 and ED-1 cells were determined by MTT assay at 0, 24, 48, and 72 h. A549 and ED-1 cells were seeded into 24-well plates at a density of 2.5 x 10^4^ (cells/well) and cultured in (**a**) 10 % fetal bovine serum (FBS) or (**b**) A549 and ED-1 cells cultured in 0, 0.5, 1, 2.5, 5, and 10 % FBS, respectively. The results were expressed as the fold change relative to the result at 0 h. All values represent the means ± S.D. of triplicate samples

The ED-1 cell line was derived from transgenic mice with wild-type human cyclin E expression in lung cancers [32, 33]. The human A549 lung cancer cell line, with constitutive cyclin E expression, is highly permissive for oncolytic Ad replication [17]. The growth properties of ED-1 and A549 were compared to understand the difference between the two cell lines. The final number of ED-1 cells increased 10 fold in 3 days, while A549 cells increased 5.6 fold (Fig. 1a). The doubling time generated from the ED-1 cell growth data in log phase was 18.60 h and A549 was 30.17 h, showing the ED-1 cell growth rate was about 1.6-fold faster than that of A549 cells. Growth curves of A549 and ED-1 cells in the presence of serum concentrations, ranging from 0 to 10 %, are shown in Fig. 1b. The number of A549 cells cultured in medium with 0 % serum only increased slightly; however, ED-1 cells still increased close to 4 fold under the same conditions. Thus, ED-1 cells grew significantly faster and exhibited less dependence on serum concentration than A549 cells.

### Murine ED-1 cells require 3-fold higher titers of Ads to achieve similar infection as human A549 cells

**Fig. 2:**
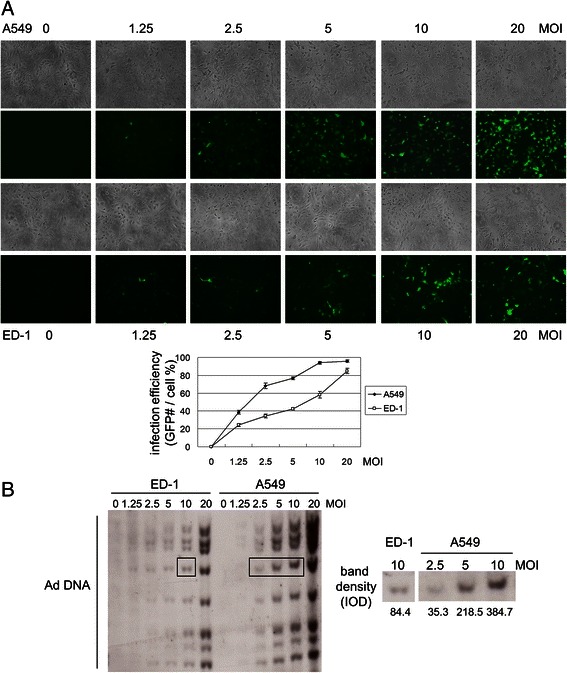
Infection efficiency of human adenoviruses on A549 and ED-1 cells. (**a**) A549 cells were cultured with 1 % FBS, and ED-1 cells were cultured with 0 % FBS at a density of 10^5^ (cells/well) and infected with increasing MOI of AdGFP after seeding for 4 h. For each infection, three random fields were taken by EVOS fluorescence microscope (AMG, Bothell, WA) at 72 h post-infection (p.i.). The numbers of GFP cells on each photo were calculated by ImageJ (US National Institutes of Health, Bethesda, MD). The numbers of GFP-positive cells were divided by total cell numbers on each photo to determine the infection efficiency. All values represent the means ± S.D. of triplicate samples. (**b**) A549 cells were cultured with 1 % FBS, and ED-1 cells were cultured with 0 % FBS at a density of 10^6^ (cells/well) in 60-mm dishes. Cells were infected with AdGFP at 0, 1.25, 2.5, 5, 10, and 20 MOI, respectively, and harvested at 24 h p.i. The DNA samples were digested with *Pst*I, and then totally loaded into the agarose gel for Southern blot analyses with Ad DNA fragments. The amounts of AdGFP entering cells were quantitated by Gel-pro Analyzer 4.0 software (Media Cybernetics, Bethesda, MD) and presented as integrated optical density (I.O.D.) values. (*Right*, magnified view of boxed section.)

To evaluate the efficiency of human Ad infection of human and murine cancer cells under a similar growth rate and a comparable density, A549 and ED-1 cells were cultured in 1 % FBS and 0 % FBS, respectively. We first evaluated the Ad infection by quantifying the number of A549 and ED-1 cells expressing GFP after infection with AdGFP (Fig. 2a). Infection with AdGFP at an MOI of 10 achieved maximum infection (>90 %) of A549 cells, while an MOI of 20 infected only 80 % of ED-1 cells, indicating poor infection of Ads in mouse cells. Considering that the activity of the cytomegalovirus (CMV) promoter used to drive GFP expression in the vector may differ in different cell lines, we also evaluated the infection efficiency by quantitating the amount of Ad DNA in cells. As AdGFP is a non-replicative virus, the amounts of AdGFP DNA inside cells represent the total viruses that entered into those cells. With the same MOI of AdGFP infection, the Ad DNA amount in ED-1 cells was lower than that in A549 cells (Fig. 2b). Yet, increasing infection MOI of AdGFP led to a concomitant increase of Ad DNA amount in both ED-1 and A549 cells, suggesting that virus entry can be adjusted by altering the infection MOI of Ads. To compare the concentration of Ad DNA in ED-1 and in A549 cells, we specifically quantitated the densities of a band of viral DNA in ED-1 cells infected with 10 MOI of AdGFP and in A549 cells infected with AdGFP at MOIs of 2.5, 5, and 10 (boxed in Fig. 2b). The algorithmic result revealed that an MOI of 10 of Ads for ED-1 cells is required to achieve a similar infection of A549 cells at an MOI of 3.5.

### Human oncolytic Ads selectively replicate in murine ED-1 cancer cells

**Fig. 3:**
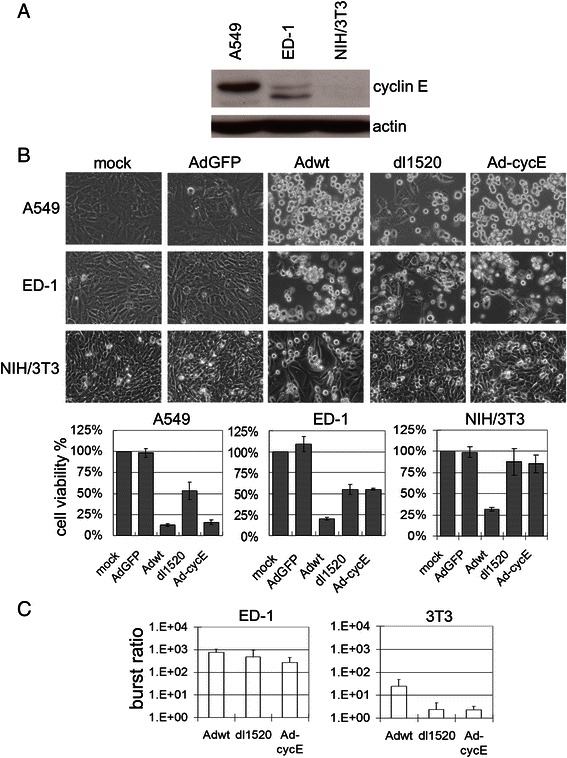
Features of cancer selectivity of human oncolytic adenoviruses on murine cells. (**a**) Cells were seeded in 60-mm dishes at a density of 10^6^ for 24 h and then collected. The cell lysates were immunoblotted for cyclin E protein and actin. Actin was used as a loading control. (**b**) Cells were mock-infected or infected with AdGFP, Adwt, dl1520, or Ad-cycE at 3.5 MOI (for A549 cells) or 10 MOI (for ED-1 and NIH/3T3 cells). Cytopathic effect (CPE) was observed at 72 h p.i. and photographed with an inverted microscope Olympus CKX41. The cell viability percentage was determined, and the values represent the means ± S.D. of triplicate samples compared with the mock-infected group. (**c**) ED-1 or NIH/3T3 cells were infected with Adwt, dl1520, and Ad-cycE at 10 MOI for 18 h or 120 h. The virus yields were determined by infection unit method and expressed as burst ratios, representing virus yields at 120 h p.i. relative to virus yields at 18 h p.i. The values represent the means ± S.D. of triplicate samples

To understand the potential of murine ED-1 cancer cells for the study of oncolytic virotherapy, we compared Ad replication in murine and human cancer cells. Murine NIH/3T3 cells generated from NIH Swiss mouse embryo fibroblasts [49] were applied here as a non-cancerous control. Relatively higher levels of cyclin E expression were detected in human A549 cancer cells, expression of cyclin E was lower in the murine ED-1 cell, but not in NIH/3T3 cells (Fig. 3a). Replication of wild-type Ad5 (Adwt), oncolytic dl1520 (ONYX-015), and Ad-cycE were evaluated. AdGFP was used as a non-replicative control. Ad dl1520 is an attenuated Ad with *E1b* deletion, which has been studied in several clinical trials [2, 35]. Ad-cycE is an *E1b*-deleted vector with its *E1a* gene controlled by the human cyclin E promoter [34]. To achieve equal infections, we chose 3.5 MOI of Ad for infection of human A549 cells and 10 MOI for murine cells in our in vitro experiments. The photographs and quantitated data of cell viability showed that mock-infection and infection with non-replicative vector AdGFP did not induce cytotoxicity (Fig. 3b). Adwt induced cytotoxicity in all cell lines. However, the two oncolytic viruses, dl1520 and Ad-cycE, induced significant cytotoxicity in both A549 and ED-1 lung cancer cells but not in non-cancerous NIH/3T3 cells. This suggests the selective cytotoxicity of oncolytic Ads for both human and murine cancer cells.

To determine whether the cytotoxicity was caused by complete virus replication in murine cells, burst assay was used to determine the virus production. Yields of Adwt, dl1520, and Ad-cycE increased over 100 fold in ED-1 cancer cells. Adwt titers also increased in NIH/3T3 cells, but dl1520 and Ad-cycE replication was strongly repressed in NIH/3T3 cells (Fig. 3c). The results indicate that Adwt can replicate in both cancer and non-cancerous murine cells; however, dl1520 and Ad-cycE can preferentially replicate in murine ED-1 cancer cells.

**Fig. 4:**
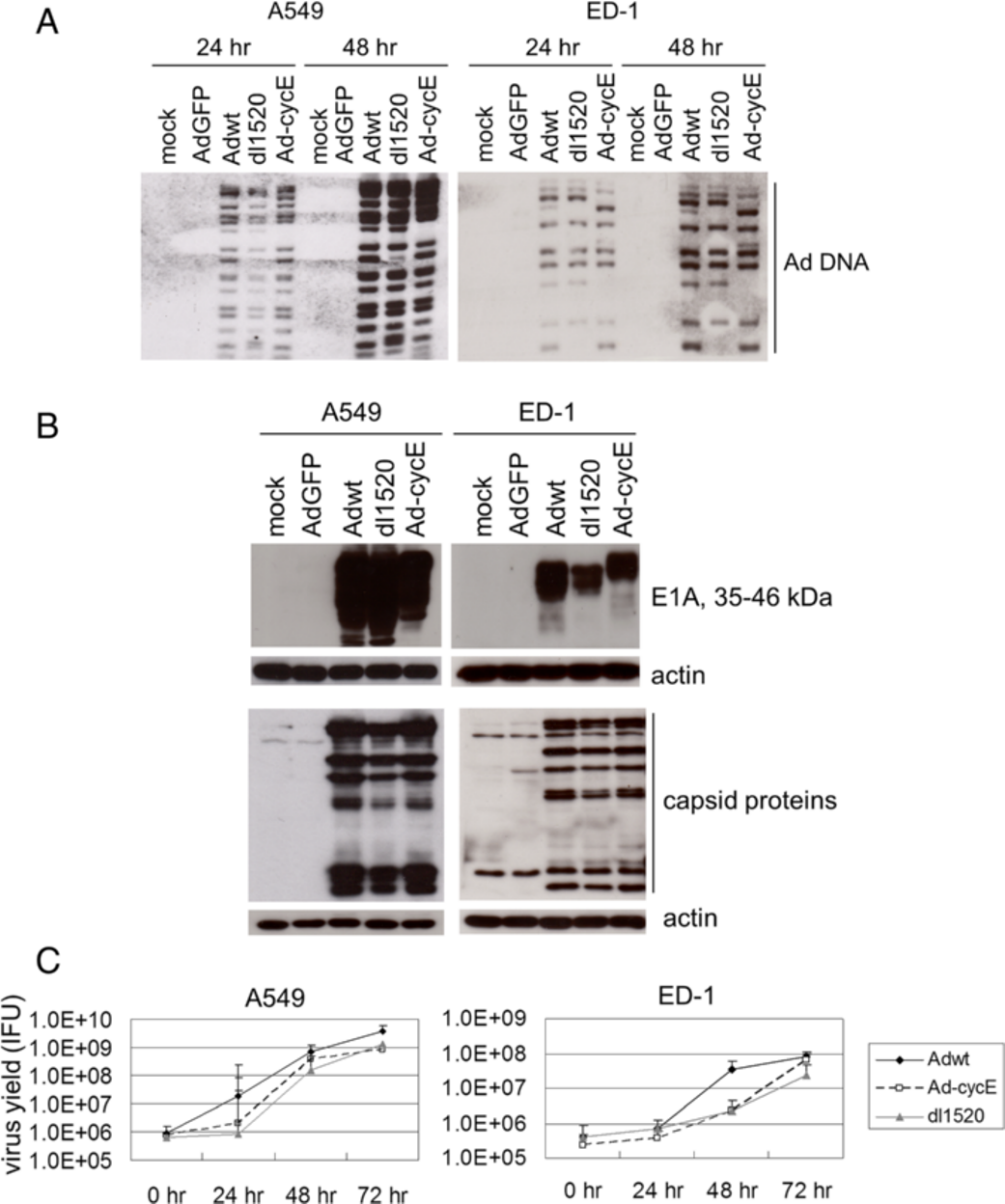
Characterization of human adenoviral replication on murine cells. (**a**) 3.5 MOI was used to infect A549 cells, and 10 MOI was used to infect ED-1 cells to achieve the similar infection efficiency. Virus DNA synthesis was determined by Southern blot analyses. Multiple bands in the range of 35–46 kDa were generated from the alternative splicing of E1A transcripts. (**b**) Cells were mock-infected or infected with AdGFP, Adwt, dl1520, or Ad-cycE at 3.5 MOI (for A549 cells) or 10 MOI (for ED-1). Cells were collected at 24 h p.i., and the cell lysates were immunoblotted for E1A protein, viral capsid protein, and actin. Actin was used as a loading control. (**c**) The virus yields were determined at 0, 24, 48, and 72 h p.i. with the infection unit method. The values represent the means ± S.D. of triplicate samples

To further characterize the properties of human Ad replication in A549 and ED-1 cells, Ad DNA synthesis, E1A expression, the production of viral capsid proteins, and the virus yields were analyzed. Southern blot analyses showed that viral DNA levels increased from 24 to 48 h post infection (p.i.) in A549 and ED-1 cells infected with Adwt, dl1520, and Ad-cycE (Fig. 4a). The level of E1A expression was examined by Western blot analyses at 24-h p.i. Ad E1A expression was only detected in the groups infected with replication-competent Adwt, dl1520, and Ad-cycE, but not in the groups mock-infected or infected with AdGFP (Fig. 4b). Consistent with the pattern of the viral early gene E1A expression, capsid protein of viral late gene production at 72 h was detected in both human and murine cancer cells infected with Adwt, dl1520, and Ad-cycE (Fig. 4b). Virus yields of human Ads in murine ED-1 cells and human A549 cells increased over the time (Fig. 4c). The titers of Adwt, dl1520, and Ad-cycE produced by A549 cell culture increased to ~10^9^ (IFU/ml) at 72 h after infection, while the virus titers produced by ED-1 were between 10^7^ and 10^8^ (IFU/ml) (Fig. 4c). Altogether, our data demonstrate that Adwt and oncolytic dl1520 and Ad-cycE can replicate in both human A549 and murine ED-1 lung cancer cells.

### Ad-cycE suppresses murine ED-1 tumor growth in immunocompetent mice

**Fig. 5:**
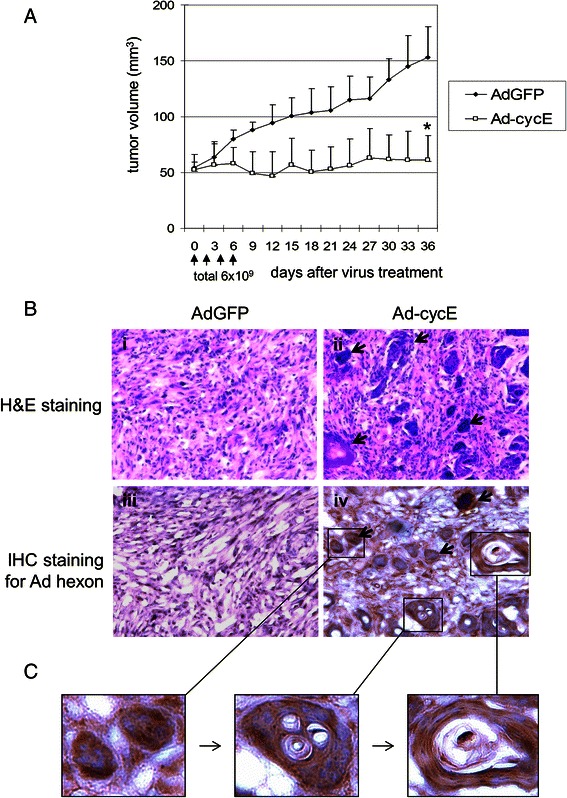
Antitumor effects of human Ad-cycE on murine ED-1 tumor in FVB mice. Mice carrying established ED-1 tumors were treated with control virus AdGFP or Ad-cycE on days 0, 2, 4, and 6. The total viral dose was 6 x 10^9^ IFU/mouse. (**a**) The vertical arrow represents each treatment. Tumor volume (V) was plotted against time and was determined by the equation V = (L × W^2^) / 2, in which L represents the length, and W represents the width of the tumor. The values represent the mean tumor volumes ± S.D. * *P* < 0.05 compared with AdGFP control group (*n* = 5), Student’s *t*-test. (**b**) H&E staining and immunohistochemical staining of tumor sections using anti-adenoviral hexon antibody. The representative photographs were taken at original magnification X200. Arrows in panel ii point to formed, condensed structures in the tumor tissues. Arrows in panel iv point to strong hexon staining. (**c**) The magnified views show the progression of pathologic morphology in the sections from mice treated with Ad-cycE

The expression of the cyclin E gene in murine ED-1 cells is under control of the human surfactant C promoter (SP-C) promoter [32]. The SP-C promoter has been used for lung epithelial cell-specific gene expression in transgenic models [50–53]. The SP-C promoter may be more active in vivo than in vitro [54]. To evaluate the effect of Ad-cycE in vivo, we subcutaneously injected murine ED-1 lung cancer cells into immunocompetent FVB mice. When tumors were approximately 50 mm^3^, the mice were intratumorally injected for a total of 4 times with total 6 × 10^9^ IFU of AdGFP or Ad-cycE. The initial reduction of tumor volumes in the Ad-cycE-treated group was observed at day 9 after the first treatment. Mice treated with Ad-cycE exhibited significant suppression of tumor growth, with 60 % reduction in the mean tumor volume as compared with mice treated with control AdGFP at day 36 after the first treatment (*P* = 0.0002, Fig. 5a).

To verify the virus replication in tumor tissue, tumors from the mice were harvested at day 7 and analyzed with histological and immunohistochemical analyses. H&E staining showed that some cancer cells formed condensed structures in the tumor tissues treated with Ad-cycE (Fig. 5b, panel ii, blue areas indicated by arrows). In contrast, such structures did not occur in the tumors treated with the control vector AdGFP (Fig. 5b, panel i). Immunohistochemical studies with the antibody against the Ad hexon protein, the classic marker of virus particles produced in cells [55], showed the expression of the viral hexon protein in tumor tissues treated with Ad-cycE, but not in the control group treated with AdGFP (Fig. 5b, panels iii and iv), indicating de novo synthesis of Ad-cycE viral late proteins. The strong hexon staining regions (Fig. 5b, panel iv, brown areas indicated by arrows) are consistent with the condensed structures showed by H&E staining (Fig. 5b, panel ii, blue areas). The magnified views further illustrated the possible progression of the pathologic morphology change in the tumor sections after Ad-cycE treatment (Fig. 5c). The results showed that Ad-cycE-infected ED-1 tumor cells were encircled in capsule-like structures and likely killed as a consequence of oncolytic Ad replication indicated by the high level expression of the viral hexon protein, forming vacuoles in the capsules. However, cells outside of the capsules were not infected by Ad-cycE and survived the treatment.

## Discussion

Development of an immunocompetent murine model for oncolytic Ad therapy is critical to accurately evaluate and improve the efficacy and safety of this approach. In this study, we have characterized human Ad replication in murine ED-1 lung cancer cells and studied oncolytic Ad therapies with ED-1 tumors developed in immunocompetent mice. Our data revealed that *E1b*-deleted Ad viruses replicated in the ED-1 cells and repressed ED-1 tumor growth in syngeneic immunocompetent mice.

Murine cells were generally considered not permissive for human Ad replication [56]. We found in this study that human Ads can infect ED-1 cells and selectively replicate in and destroy the murine cancer cells. The cross-species infection of human Ad in ED-1 cells can be mediated via the homologous Ad receptor CAR on murine cells [57], coreceptors such as integrins and heparan sulfate glycosaminoglycans, or other unknown receptors. We observed that the virions of oncolytic Ads were produced in murine cancer ED-1 cells, but not in non-cancerous murine NIH/3T3 cells. Obviously, the entire human Ad life cycle is completed in ED-1 lung cancer cells, but restricted in NIH/3T3 cells. It has been reported that human Ads normally undergo abortive replication in murine cells [58, 59]. Virus yields of Ad2 and Ad5 in 3T3-Swiss and BALB/c 3T3 cells were reduced 3 to 5 logs compared to virus production in human cells [60, 61] and no infectious virus particles of Ad12 were detected in murine 3T3 (embryo) and L (connective tissue) cells [59, 62]. The block of human Ad replication in murine cells may be related to the low infectivity [63], unstable and reduced DNA synthesis [59, 61, 64], abortive expression of late proteins, or the defective assembly and maturation at an later stage of viral replication cycle [59, 60, 62]. Previously, we observed that human Ads induced cytotoxicity in Chinese hamster ovary (CHO) cells; however, there was a lack of late protein production that prevented infectious virion production (Cheng and Zhou, unpublished data). While the exact mechanism(s) supporting the complete multiplication process of human Ads in murine cancer ED-1 cells remains to be investigated, it is tempting to speculate that the human cyclin E overexpression in the cell may play an important role. The human cyclin E gene in ED-1 cells was validated by polymerase chain reaction (PCR) [33] (Additional file 1: Figure S1A). We also performed single-cell cloning and validated the level of cyclin E protein expression in these subcloned ED-1 cells. The cyclin E proteins were detected at 47 kDa and 37 kDa [32, 65] in parent ED-1 and all subclones (Additional file 1: Figure S1B). Any conclusions, though, cannot exclude the possibility that the cyclin E proteins detected in the cultured ED-1 cells may be murine proteins, since the human cyclin E gene is under the control of the SP-C promoter that may be restricted in cultured cells [54]. In our study, we observed that the cyclin E protein produced in ED-1 cells was much lower than that observed in A549 cells (Fig. 3a), that may indicate the repression of the cyclin E expression caused by the restricted SP-C promoter in ED-1 cells.

The transgenic cyclin E expression in ED-1 cells may directly and indirectly affect the multiplication process of oncolytic Ads. Under the normal condition, cyclin E regulates cell cycle progression, DNA replication licensing [21, 22, 66], centrosome duplication [23, 24], and E2F activation [67]. Cyclin E overexpression may cause cell overgrowth and thus increase the accumulation of mutations associated with tumorigenesis. Consequently the alternations of the growth rate and genetic mutations may create a suitable environment to loose cellular restriction to viral propagation. Also, the excess cyclin E may directly endorse virus replication. We previously demonstrated that human Ad replication relies on cyclin E induction in cells after viral infection [17]. Ad-induced cyclin E turns on the pRb/E2F pathways by activating CDK2 [19]. It is possible that human Ad replication in murine ED-1 cells may be associated with cyclin E dysregulation or cell cycle alterations occurring in the carcinogenesis caused by the transgenic cyclin E expression.

We have investigated the antitumor efficacy of Ad-cycE with murine tumors in immunocompetent FVB mice. Interestingly, we identified that Ad-cycE-infected cancer cells located in the specific areas where the clusters of cells were encircled in capsule-like structures in tumors (Fig. 5c). The capsule formation is likely associated with Ad oncolytic replication because tumors treated with the control non-replicating AdGFP did not exhibit such structures. We found that cells inside the capsules died and formed vacuoles, but cells outside were not infected and survived the treatment. Thus, the capsule structures developed in tumors after Ad-cycE infection may prevent viruses from spreading to the entire tumor. In a previous clinical study, oncolytic Ads were observed in clusters of 5–20 cells after intratumoral administration, indicating that Ad spread in tumors is restricted [68]. Viral spread within solid tumors is limited, and usually is around the site of injection after intratumoral delivery [69, 70]. The movement of viruses through tumors is likely impeded by the dense tumor extracellular matrix [1]. Hyaluronan is a key component of the tumor extracellular matrix. With an oncolytic Ad expressing hyaluronidase to degrade this kind of important structural element of the ECM, Guedan et al. (2010) showed that the virus distribution could be improved in a human melanoma xenograft model [71]. Our results indicate that the capsule structures may be formed as a consequence of active tumor reactions to Ad replication to prevent progeny Ad virions from spreading the infection to the rest of the cancer cells in tumors.

It is possible that the capsule structures may be also associated with immune responses of FVB mice to Ad replication. Hallden et al. (2003) reported that Ad5 significantly induced intratumoral inflammatory cell infiltration, including macrophage and CD8(+) lymphocytes [9]. The induction of non-specific or specific antitumor immunity has been reported as one of the mechanisms to mediate tumor cell lysis [72, 73]. The detailed mechanism by which the capsules formed in tumors requires further study. The immune system of the ED-1 animal model may have multiple effects on oncolytic virotherapy. Further studies will clarify these immune-mediated effects, such as the role of the immune cell infiltration into the tumors on Ad spread in tumor. The ED-1 animal model as a preclinical system will also benefit the development of future strategies to enhance viral penetration and spread within solid tumors.

## Conclusion

Our results showed that murine ED-1 cancer cells are permissive for human Ad replication, and Ad-cycE significantly represses ED-1 tumor growth in immunocompetent FVB mice. Such a model with the unique background of cyclin E overexpression can provide a suitable in vivo environment for researchers to study oncolytic Ad replication in detail. Moreover, the capsule structures formed in tumors may prevent viruses from spreading to the entire tumor. The ED-1 model may provide an opportunity to recapitulate clinical phenomena and challenges for studies of virus spread in tumors and the interactions between Ads and immune system. An orthotopic tumor model based on this system can be established to look at tumor growth and therapeutic efficacy in the context of the lung microenvironment and thus provide a valuable system to study oncolytic virotherapy.

## Additional file

Additional file 1: Figure S1. Characterization of cyclin E background in murine ED-1 cells. (**a**) The genomic DNA was isolated from ED-1 cells, and PCR was used to detect cyclin E with sense primer 5'-TTG GCT ATG CTG GAG GAA GTA-3’ and antisense primer 5'-AGT GCT CTT CGG TGG TGT CAT-3’. (**b**) The cell lysates from parent ED-1 and single-cell clones were immunoblotted for cyclin E proteins. (TIFF 289 kb)

## Abbreviations

Ad: Adenovirus
CTLs: Cytotoxic T lymphocytes
CDK2: Cyclin-dependent kinase 2
FBS: Fetal bovine serum
DMEM: Dulbecco’s Modification of Eagle’s Medium
GFP: Green fluorescent protein
MTT: 3-(4,5-dimethylthiazol-2-yl)-2,5-diphenyltetrazolium bromide
OD: Optical density
I.O.D.: Integrated optical density
CPE: Cytopathic effect
MOI: Multiplicity of infection
p.i.: post infection
PBS: Phosphate-buffered saline
IFU: Infectious unit
O.C.T.: Optimal cutting temperature compound
H&E: Hematoxylin-eosin
IHC: Immunohistochemical staining
Adwt: Wild-type adenovirus
S.D.: Standard deviation
CAR: Coxsackievirus and adenovirus receptor
CHO: Chinese hamster ovary
pRb: Retinoblastoma protein
