# Supplementary figures and images for: Oncolytic adenovirus targeting cyclin E overexpression repressed tumor growth in syngeneic immunocompetent mice
Source: BMC Cancer. 2015 Oct 16;15:716. doi: 10.1186/s12885-015-1731-x (PMC4609153; doi:10.1186/s12885-015-1731-x)

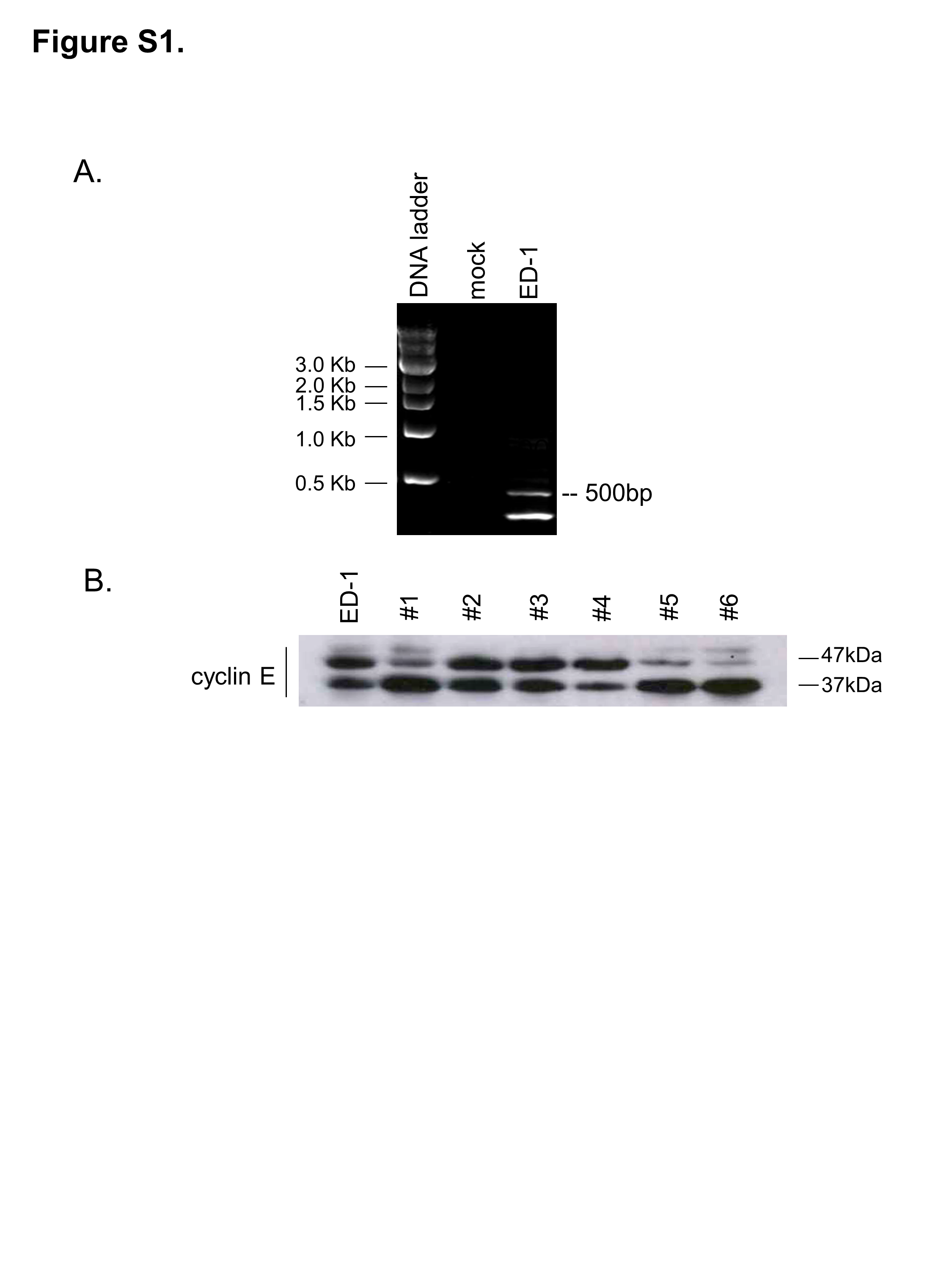

Supplement: Additional file 1: Figure S1. — Characterization of cyclin E background in murine ED-1 cells. (a) The genomic DNA was isolated from ED-1 cells, and PCR was used to detect cyclin E with sense primer 5'-TTG GCT ATG CTG GAG GAA GTA-3’ and antisense primer 5'-AGT GCT CTT CGG TGG TGT CAT-3’. (b) The cell lysates from parent ED-1 and single-cell clones were immunoblotted for cyclin E proteins. (TIFF 289 kb) [file 12885_2015_1731_MOESM1_ESM.tiff]
